# The Colt Device for Treating Thoraco-Abdominal Aneurysms - Concept and Clinical Results

**DOI:** 10.31083/j.rcm2307239

**Published:** 2022-06-24

**Authors:** Piotr Szopiński, Eliza Pleban, Jarosław Iwanowski

**Affiliations:** ^1^Clinic of Vascular Surgery, Institute of Hematology and Transfusion Medicine, 02-776 Warsaw, Poland

**Keywords:** thoraco-abdominal aortic aneurysm, endovascular repair, new endovascular multibranch stent graft, new device

## Abstract

**Objective:**

To report results of application a new stent graft design for the treatment of patients with thoraco-abdominal aneurysms (TAAAs), which was co-invented by a vascular surgeon. This is a retrospective observational study.

**Methods:**

The Colt is a self-expanding stent graft, composed of nitinol metal stents creating a special exoskeleton with asymmetric springs covered with polyester material. The Colt device offers some advantages over existing stent graft options. The main body is available in two different diameters on both ends and in three different lengths. It has four branches pointing downward and coming from the main stent graft at two levels. It offers the physician an opportunity to decide which branch to choose for the target vessel. It may be implanted alone or extended proximally and distally. Balloon expandable and/or self-expanding stent grafts are used to create the visceral branches. In complex extensive aneurysms, the procedure is divided into two or three stages to minimize the risks of spinal cord ischemia.

**Results:**

Between August 2015 and December 2021, twenty-two Colt stent grafts were implanted in twenty males and two females (aged 56–81) with TAAAs (eight Type II; twelve Type III; two Type IV). The mean aneurysm diameter was 73.4 mm (range 64–83). All patients were asymptomatic. Eighty-five target vessels were reconstructed using either self-expanding or balloon-expandable stent grafts. Fourteen bifurcated, six custom-made tubes and two aortouniiliac (AUI) stent grafts were used as distal extensions to the Colt device. Completion angiography revealed no type I endoleaks. Five patients had Type II endoleaks which were treated conservatively. There were no intraoperative deaths. One patient died on the 7th postoperative day from multiorgan failure. We did not observe any other complications within 30 days after implantation. One patient died from Covid-19 two months after discharge. Follow-up ranged from three to 75 months. There was no migration or dislocation of the docking station or proximal and distal extensions. All Colt device prostheses remained patent, however, two branches leading to the coeliac trunk were found occluded at the time of the 12-month CTA, without any symptoms. In two patients, there were late problems with three renal bridging stent grafts. One of the Type II endoleaks resolved spontaneously after one year, while four others remain under observation. No patient had an increase in sac diameter.

**Conclusions:**

Results from the current series are promising. The Colt stent graft can be applied to a large variety of TAAA anatomies, which may facilitate the development of new “off-the-shelf” devices in the future.

## 1. Introduction

Although thoraco-abdominal aortic aneurysms (TAAA) are relatively uncommon in clinical practice, if left untreated up to 74% of patients experience aneurysm rupture [1, 2, 3, 4]. Currently, there are three treatment options: surgical, endovascular and conservative. Open surgical repair is effective, but with a relatively high mortality rate (up to 20%) it may only be justified in otherwise fit patients with larger aneurysms [5, 6]. Endovascular aneurysm repair (EVAR) offers an alternative treatment. The role of fenestrated and branched stent grafts in the management of TAAA has grown owing to its low perioperative mortality rate of about 10% and excellent short-term and mid-term results [7, 8, 9]. Commercial custom-made devices, however, involve a 6–12 week delay for manufacture. Consequently, alternative strategies (periscope, chimney or physician-modified stent grafts) have been developed for use in urgent cases [10, 11, 12, 13]. Currently, “off-the-shelf” multi-branched endovascular prostheses are available. However, due to anatomical limitations, they are not applicable for use in all patients [10]. A recently introduced inner branch technology—the E-nside TAAA system—may be useful in complex anatomy, where application of fenestrations or side-arm branches can be challenging [14].

The purpose of this retrospective analysis is to evaluate the safety and feasibility of a new custom-made E-xtra design stent graft device for treating TAAA co-invented by a vascular surgeon.

## 2. Materials and Methods

### 2.1 The Concept 

The first aim was to create a “docking station”, which would become the main module of the entire prosthesis. Once established, proximal and/or distal stent graft extensions can be inserted to treat any concurrent thoracic or abdominal aneurysm. The docking station was specifically designed to allow downward catheterization of visceral vessels. Bench and fatigue testing were performed on all components of the new stent graft, as well assessment of pull and displacement forces between the branches and covered stents. These test results showed ≥1.1 N with no oversizing over a sealing length of 15.84 mm. Finally, X-ray tests and simulation of the procedures were performed with the use of an *in vitro* aneurysm model.

### 2.2 The Device Description

**Fig. 1.**
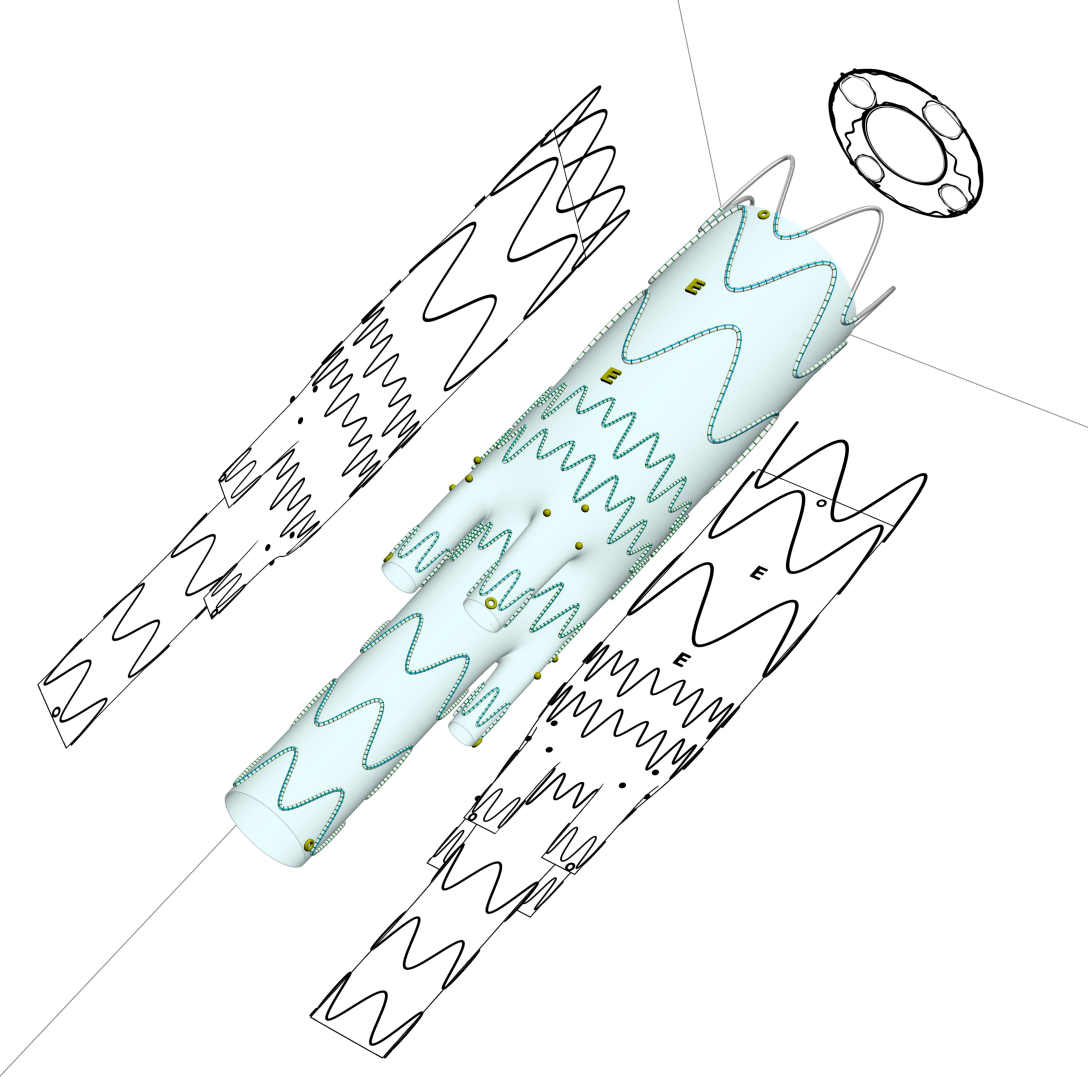
**Technical drawing of the Colt device with sections with the positioning of the markers**.

**Fig. 2.**
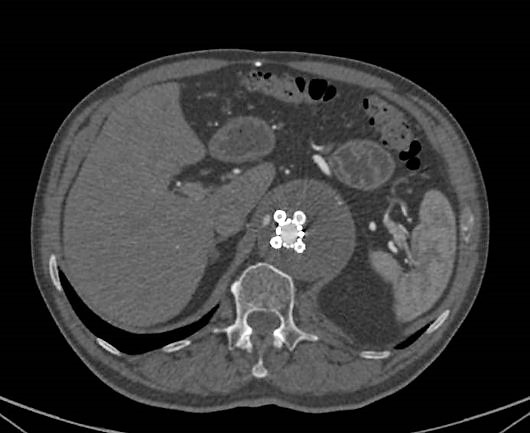
**In CTA cross-section the docking station resembles the revolving chamber of a Colt handgun**.

The Colt stent graft (Jotec GmbH, Hechingen, Germany) is a self-expanding custom-made E-xtra design device composed of nitinol metal stents of different dimensions, creating a special endoskeleton, which is sewn onto the multifilament polyester (PET (polyethylene terephthalate)) graft fabric. In the branched segment, asymmetric springs are used, significantly increasing the radial force and reducing the folding of the textile. The device is equipped with a combination of different radiopaque gold markers (two rings anterior and posterior at the proximal edge of covering material, two E-markers on the front surface of the main body, three dots at the entrance of the side-branch creating a triangle, one circle marker at the distal end of each branch and two rings placed on both lateral sides at the distal edge of the covering material), increasing the visibility of each feature (Fig. 1). The stent graft consists of a tapered tube the diameter of which is 33 mm or 36 mm proximally, decreasing to 16 mm or 18 mm distally. The length of the covered segment of the stent graft is 122 mm, 175 mm or 200 mm. It has two large upper branches located at 10 and 2 o’clock (branches A and B; 8 mm in diameter, 19 mm in length) and two smaller caudal branches located at 8 and 4 o’clock (branches C and D; 6 mm in diameter, 22 mm in length). All four branches are oriented downward, in contrast to some other devices, e.g., Zenith t-Branch, whose side branches are positioned at an angle. This construction gives the operator the opportunity to decide which branch to use for which target vessel, even during the procedure. The upper anterior branches are used to cannulate the coeliac trunk and superior mesenteric artery (SMA), while the lower branches are used for cannulating renal arteries. Each branch is connected with the main graft with an oval-shaped patch and supported with a separate asymmetric spring as an exoskeleton. On the inside the orifices are elliptic. In cross-section, the stent graft resembles the revolving chamber of a Colt handgun (Fig. 2). For better fixation, a free*-*flow bare spring has been added to the proximal end. The device is delivered through a 24-F system.

### 2.3 Planning and Sizing

A preoperative computed tomography angiography (CTA) is acquired using 1 mm slice thickness. Procedure planning and device sizing are performed using a dedicated three-dimensional vascular imaging workstation (OsiriX Pro) with centerline luminal reconstruction. The clinician receives an initial plan sketch of the entire procedure with drafts of all the endoprosthesis components. After final approval, the implant can be manufactured within three weeks.

### 2.4 Anatomical Requirements for the Colt Stent Graft

The following criteria determine the possibility of Colt device implantation:

- landing/sealing zone of 30–40 mm in the descending aorta or extension/preparation of neck with thoracic stent graft

- perfused aortic diameter ≥26 mm

- diameter of visceral/renal arteries ≥4 mm and ≤10 mm

- length of visceral/renal arteries ≥20 mm

- adequate access vessels

### 2.5 Implantation Technique

**Fig. 3.**
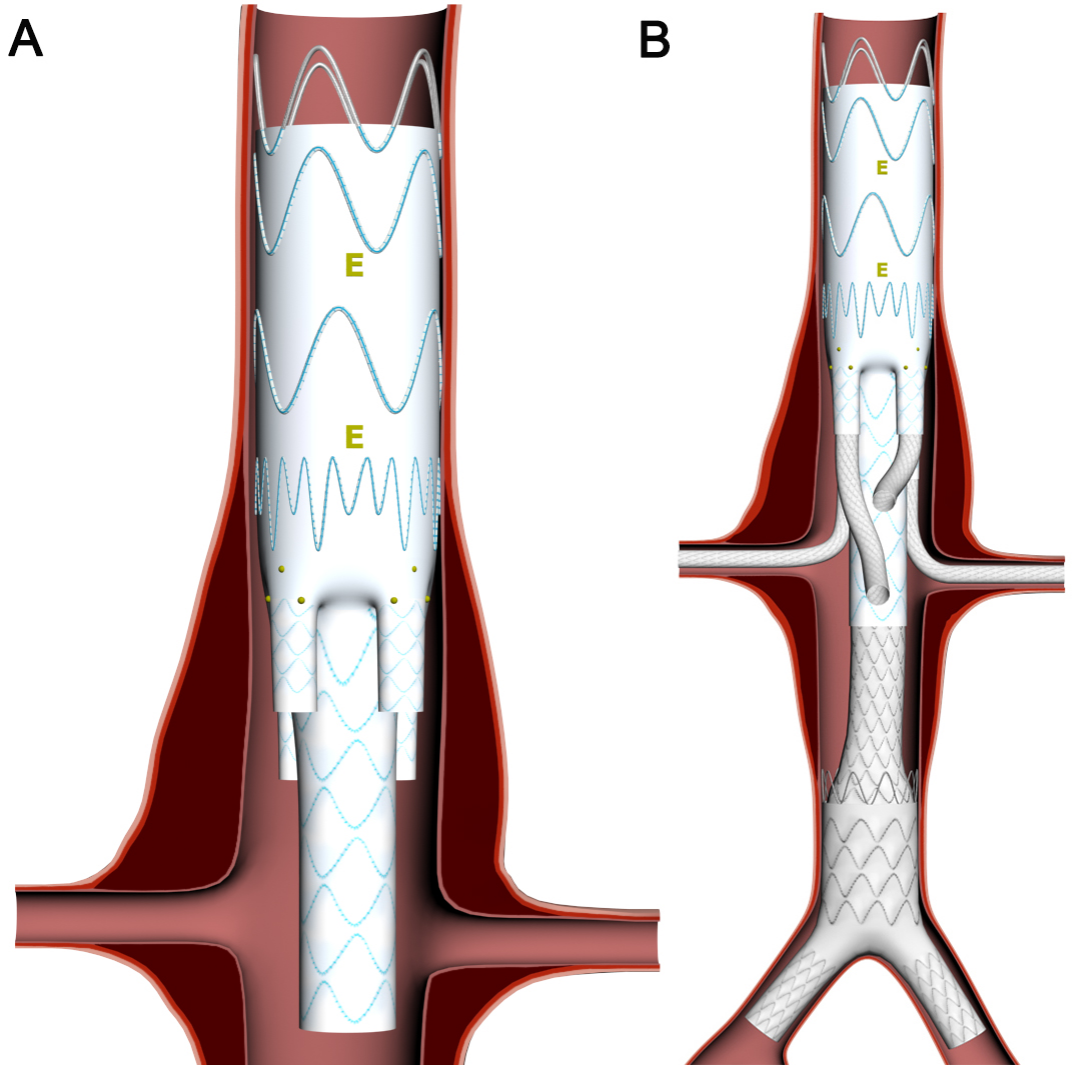
**The steps of the Colt device implantation**. (A) The Colt device is positioned with the ends of the lower branches at the level of coeliac trunk orifice. Note that the docking station may be implanted alone or with the thoracic stent graft as a proximal extension. (B) The Colt device is fully deployed with all four bridging stent grafts and extended with bifurcated endoprosthesis. Note that tube distal extension is also possible.

All implantations were performed in a hybrid room equipped with a Siemens Artis Zee system (Siemens Medical Solutions, Malvern, PA, USA) by the same team of two vascular surgeons, one interventional radiologist, a dedicated anesthesiologist and trained scrub nurses. All procedures were conducted under a combination of spinal and general anesthesia. In accordance with the adopted protocol, the patients are positioned supine, with both arms or only the left arm abducted and the imaging unit oriented from the head of the table. Both femoral arteries and one axillary artery are exposed. An arterial catheter is used to measure the mean arterial blood pressure in the radial or brachial artery, which is to be maintained >90 mmHg. The patients are systemically heparinized with an intravenous bolus of heparin (80–100 units/kg) administered immediately after femoral and brachial access is established. Activated clotting time (ACT) is checked every 30 minutes and additional heparin is administered if the ACT falls below 250 seconds. Renal function is monitored. Diuresis is induced using intravenous furosemide. After the first lateral angiographic view, the coeliac trunk is identified and catheterized via the axillary artery with a soft 0.018”, 400-cm-long Terumo Radifocus Guide Wire M (Terumo Europe N.V., Leuven, Belgium), and used as the primary reference point. The Colt device is then advanced from the femoral access along an axillo-femoral through-and-through guidewire. The 12-F 45-cm-long Flexor sheath (Cook Medical, Inc., Bloomington, IN, USA) is introduced along the same guidewire from the axillary artery to the descending aorta in order to minimize manipulation within the arch. The Colt device is positioned with the markers of its lower branches at the level of the coeliac trunk orifice. Next, the docking station is partially deployed to open the main body and the two shorter branches for the coeliac trunk and SMA, with the proximal uncovered segment closed. The coeliac trunk and SMA are then cannulated and peripheral stent grafts are delivered through a 6-F or 7-F Terumo Destination sheath (Terumo Medical Corp., Elkton, MD, USA) introduced via 12-F sheath (65-cm-long for the left side or 90-cm-long for the right side access) using Rosen guidewires (Cook Medical, Inc., Bloomington, IN, USA) and implanted one-by-one. The renal side branches are then cannulated and stent grafts were inserted into the renal arteries. Check angiograms are mandatory after cannulation of each target vessel and following deployment of the peripheral stent graft. For side branches, both self-expanding and balloon-expandable stent grafts may be used with oversizing of 1–2 mm and 0–1 mm respectively. When a balloon-expandable stent graft is used, flaring of the proximal end should be done. Sometimes when the distance between the proximal part of the branch and the desired landing zone in the target vessel is too long, it is necessary to implant two peripheral stent grafts as those of appropriate lengths are not always available. In such cases, aiming at better stabilization, we implant self-expanding stents as an internal framework (relining technique). There is a possibility of stepwise release of the “docking station”, which simplifies the cannulation of upper side-branches, by reducing the number of open channels. Finally, the Colt is fully deployed (Fig. 3). Depending on the anatomy of the aneurysm, proximal and distal extensions can be planned as staged interventions in order to minimize the risk of neurological complications. For Types I and II TAAA, it is essential to start the procedure by implanting a thoracic stent graft in order to provide a proximal landing zone for the Colt branched device. The procedure should then be followed by the implantation of a tube or a bifurcated aortic stent graft. It is our policy to adopt a planned staged approach in all cases of Type II TAAA, with at least three-week intervals between stages to minimize the risks of spinal cord ischaemia (SCI).

## 3. Results

Between August 2015 and December 2021, twenty-two Colt stent grafts were implanted into twenty males and two females, (aged 56–81 years) who had presented with TAAAs (eight Type II; twelve Type III; two Type IV). The mean aneurysm diameter was 73.4 mm (range 64–83 mm). All patients were asymptomatic. Four patients had previously undergone open repair of an infra-renal abdominal aortic aneurysm with implantation of tube prosthesis. Detailed patient characteristics are presented in Table 1.

**Table 1.** **Preoperative characteristics of patients who underwent Colt device implantations (n = 22)**.

Demographics and co-morbid conditions	
Age (years; mean (range))	68.46 (56–81)
Male sex	21 (90.9%)
Hypertension	17 (77.2%)
Coronary artery disease	10 (45.4%)
Heart failure (AHA II)	6 (27.2%)
Pulmonary disease	5 (22.7%)
Cerebrovascular disease	2 (9%)
Diabetes	8 (36.3%)
Current or past smoker	7 (31.8%)
Connective tissue disease	0 (0)

**Fig. 4.**
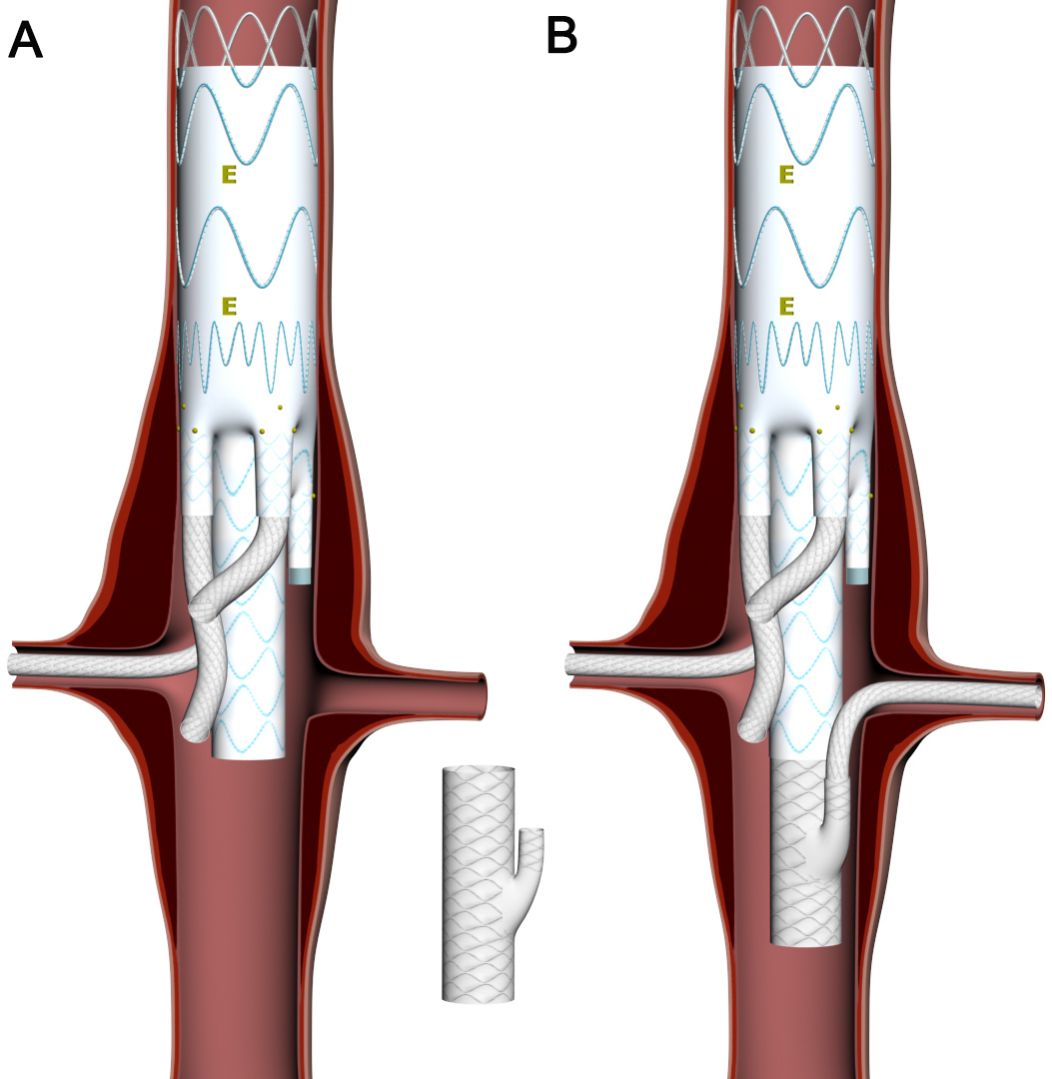
**Images showing the concept of the RRM**. (A) The renal branch of the Colt device is closed with occluder and the RRM is prepared for implantation. (B) The RRM is implanted as the Colt distal extension and its upward branch is connected with the left renal artery.

In three cases, the introduction system was delivered through a 10 mm Dacron conduit sutured to the right external iliac artery. In six cases, a proximal thoracic stent graft was deployed during the first stage of the procedure. In three cases, there was a need to modify the initial deployment strategy due to problems with the cannulation of the left renal artery. A modified custom-made stent graft with upward oriented (cephalad) branch compatible with the Colt device was implanted (Fig. 4). In another patient, the left gastric artery arose directly from the aorta (above the coeliac trunk) and this vessel was embolized at the beginning of the procedure in order to prevent a post-operative endoleak. Eighty-five target vessels were reconstructed using either the E*-*ventus BX (Jotec GmbH, Hechingen, Germany), Viabahn (Gore & Associates, Flagstaff, AZ, USA), Advanta V12 (Atrium Medical, Maquet Getinge Group, Hudson, NH, USA), Gore Viabahn VBX (W. L. Gore & Associates, Flagstaff, AZ, USA), LifeStream (Bard Peripheral Vascular, Inc, Tempe, AZ, USA) or FluencyPlus (Bard Peripheral Vascular, Inc, Tempe, AZ, USA) stent grafts. One patient had previously undergone left side nephrectomy for cancer. One renal artery was found to have become occluded in between the two stages of the procedure and one was sacrificed during the procedure because of its difficult anatomy (4 mm in diameter, severe tortuosity and almost immediate division into branches) which made its cannulation impossible from either antegrade or retrograde approaches. Fourteen bifurcated, six custom-made tubes and two AUI stent grafts were used as distal extensions to the Colt device. The remaining two Colt devices were implanted without any proximal or distal extensions. The length of the descending aorta coverage ranged from 62 to 240 mm (mean 118.4 mm). Completion angiography revealed no type I endoleaks. Five patients had Type II endoleaks, which were treated conservatively.

The mean time for the Colt implantation procedure (from introduction of the main stent graft to implantation of the last branch) was 227 minutes (range 195–297). Median blood loss was 420 mL (300–850). The median contrast volume used for Colt device implantation was 236 mL (range 20–355), while the median fluoroscopy time was 72 minutes (range 54–111), and the range of radiation dose was 1720–7378 mGy/$m^{2}$. All patients remained for at least one day in the intensive care unit after each procedural stage, according to hospital protocol. The mean hospital stay was six days. No symptoms of paraparesis/paraplegia were observed. Three hematomas after axillary access required surgical evacuation. There were no intraoperative deaths. One patient died on the 7th post-operative day due to multi-organ failure. All visceral branches and the main stent graft remained patent in this patient, confirmed by autopsy. We did not observe any other complications within 30 days after implantation. One patient died from Covid-19 two months after discharge.

**Fig. 5.**
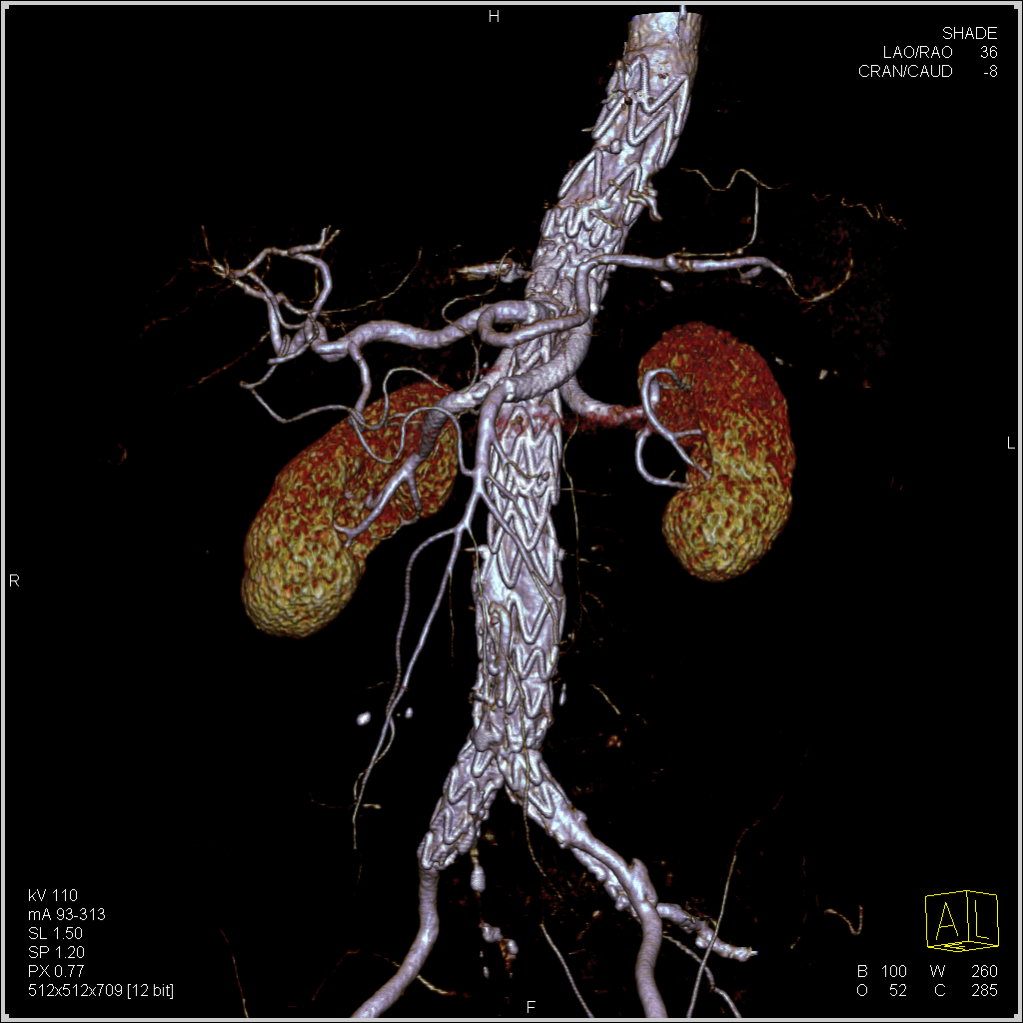
**Postoperative control CTA performed 12 months after the procedure**. The docking station with all patent branches and bifurcated distal extension.

Follow-up ranged from three to 75 months (mean 46.9). There was no migration or dislocation of the docking station or proximal and distal extensions (Fig. 5). All Colt device prostheses remained patent. Two branches leading to the coeliac trunk were found occluded at the time of the 12-month CTA, without any symptoms. In three patients, there were late problems with three renal bridging stent grafts. In one patient, a dislocated balloon-expandable stent graft positioned in the right renal artery was successfully reconnected using a Viabahn endoprosthesis. In the second patient, kinking at the level of two overlapping balloon-expandable stent rafts on the right side was treated with the insertion of an EverFlex stent (Medtronic, Minneapolis, MN, USA) after three years. In the third patient, restenosis was noted at the distal edge of the Viabahn stent graft after 3 years and was successfully treated with the insertion of an EverFlex stent. One of the Type II endoleaks had resolved spontaneously at one year, while four others remain under observation. No patient had an increase in sac diameter.

## 4. Discussion

There are limited options for treating high-risk patients with TAAA in the acute or subacute setting. Even in high volume centers, open repair is associated with mortality rates of 5–15%, increasing to 20–40% in emergency situations [15, 16, 17, 18]. Parallel techniques, such as the sandwich technique, offer an endovascular alternative. The main remaining problems with the use of this technique are the complexity of this off-label procedure, the development of type Ia endoleaks through the gutters and the risk of visceral vessel loss in follow up [11, 12, 19]. Over the last decade, the use of fenestrated/branched prostheses has gained wider acceptance as a valid approach for the management of TAAA. Expert centers have reported low perioperative morbidity and mortality rates and excellent mid-term results [7, 9].

Several “off-the-shelf” stent grafts have been introduced into the market in the last decade: Zenith t-Branch TAAA stent graft (Cook Medical, Bloomington, IN, USA), EXCLUDER thoracoabdominal branch endoprosthesis (TAMBE, W.L. Gore & Associates Inc, Flagstaff, AZ, USA), Valiant modular branched graft (Medtronic, Minneapolis, MN, USA) and E-nside TAAA Multibranch Stent Graft System (Jotec GmbH, Hechingen, Germany) with inner branches, which received the CE mark in December 2019 [15, 16, 17].

It is important to underline that at the time of the first implantations of the Colt device, the only commercially available device was the Zenith t-Branch, which was approved in Europe in 2012. The anatomical feasibility of the t-Branch prosthesis has been assessed in a few studies, which revealed that variations in target vessel configuration were the main limitation to its widespread use [18, 20]. The t-Branch device alone is only applicable in about 50% of TAAA cases, with greater suitability in staged procedures [10, 21, 22]. In the recent study by Ferreira *et al*. [23], the authors increased the anatomic suitability of the t-Branch to more than 80% of all elective and urgent thoracoabdominal aortic aneurysm cases by introducing adjunctive maneuvers and techniques outside the instructions for use (IFU), such as device modifications. The ideal off-the-shelf stent graft should be characterized by wide anatomical applicability, ease of implantation and reproducible outcomes.

We agree with the observations by Chuter *et al*. [24] that “up-going” (cephalad) renal artery branches fare poorly. They are prone to occlusion, and even when they remain patent, the size and function of the kidney often decline. Therefore, we directed all four parallel branches in the Colt device downwards. The Colt device has two 8 mm branches dedicated for cannulating and stenting the coeliac trunk and SMA and two 6 mm branches for the renal arteries originating from the main stent graft at two levels. It offers the physician the opportunity to decide which branch to choose for the target vessel, which makes the endograft more universal.

There are no recommendations as to which kind of bridging stent graft is ideal for the insertion into the visceral branches. Both self-expanding and balloon-expandable covered stents are available, but all are implanted outside IFU [25, 26, 27]. In our series both types of covered stents were implanted depending on the patients’ anatomy and their current availability. Initially, we preferred to use E-ventus, a balloon-expandable stent graft, because of its good conformability and satisfactory fluoroscopic visibility, which facilitates accurate placement. Moreover, thanks to its construction, the branch can be easily fixed into position by flaring the proximal stent graft with the same balloon that was used for its opening, which reduces the number of required maneuvers. Stability of the connections was confirmed in preclinical tests. The disadvantage of the E-ventus stent graft is its maximum length of 57 mm, which requires the use of at least two overlapping prostheses, which may consequently increase the risk of late disconnection. From the moment we found one disconnection of the E-ventus stent grafts in our fifth case, we routinely used a self-expanding stent as relining when we implanted two or more balloon-expandable grafts. In cases of unfavorable renal artery anatomy, we used the Viabahn stent graft because of its excellent flexibility. When the VBX appeared on the market with a maximum length of 79 mm it became our first choice for a bridging stent graft because of not only its length but also its conformability [28, 29]. Usually, we do not reinforce stent grafts with self-expanding bare stents unless kinking has been evident or in case of a short landing zone of the target vessel (<20 mm). A similar strategy has been presented by other authors [26, 30, 31]. We used Lifestent as a relining support for Viabahn stent grafts in one case because of the long distance between the branch and the orifice of the left renal artery. In three of our patients we encountered difficulty with left renal artery cannulation. We implanted a custom-made extension with an upward branch followed by the occlusion of the renal branch in the main graft with an occluder. Following our experience with the first patient, we were prepared with another custom-made device with an upward branch in the second case expecting some difficulties due to challenging anatomy. From then on, we were always equipped with a renal rescue module (RRM) for each Colt implantation, ready for use whenever difficulties occur. In our group all splanchnic arteries, except the above-mentioned renal arteries engaged in an RRM, were cannulated antegrade. Although retrograde access with the use of steerable sheath has been described in the literature, we did not use this technique in any of the cases in our series [32]. Lucatelli *et al*. [33] in their article describing 49 cases treated with E-xtra design engineering endografts from Jotec GmbH state that retrograde branches should be considered as a safe alternative in all cases not suitable for antegrade branches. When the Colt device is chosen as the main branched device, it is possible to plan the procedure with the use of an RRM, which could be difficult when the Zenith t-Branch is chosen.

Spinal cord ischemia following open and endovascular treatment of TAAA ranges from 3%–15% and significantly worsens perioperative morbidity and mortality [18, 23]. In the medical literature there is no explicit evidence that planned cerebrospinal fluid drainage can protect the patient from this potentially fatal complication so we think that a staged repair makes sense [34]. For that reason, we do not routinely use cerebral spinal fluid drainage but we pay careful attention to ensure that mean blood pressure is >90 mmHg and that there is adequate blood supplementation during the procedure. In cases where the TAAA involves an extended part of the descending aorta, we plan a two-staged procedure to allow time for collateralization to the spinal cord, as described by Luozzo *et al*. [35]. However, the use of a thoracic component requires a staged procedure. There is no evidence that staging the procedure augments the risk of aneurysm rupture. We agree with the conclusions of O’Callaghan *et al*. [36] that staged repair appears to protect against SCI and to enhance overall survival in patients undergoing extensive aortic repair. Our strategy also involves removal of the introduction system from the common femoral artery as quickly as possible to restore the blood flow to the lower limb and the pelvic circulation. This is usually after successful cannulation and peripheral stent graft implantation into two upper branches of the Colt, which guarantees the stabilization of the main body. A very interesting technical solution was presented by Kasprzak *et al*. [37] describing the concept of temporary aneurysm sac perfusion (TASP) and second stage side branch completion to prevent severe SCI and paraplegia after branched endovascular aortic repair.

A promising novel neuroprotective strategy was introduced by Etz *et al*. [38] who developed a minimally invasive method of selective segmental artery endovascular coil embolization as a first stage before entirely endovascular extensive TAAA repair to precondition the arterial paraspinal collateral network.

The current 34 mm diameter of the t-Branch system limits its use as a standalone device, because it requires off-the-shelf proximal extensions [31]. The ideal proximal landing zone for the t-Branch device is 24–30 mm (outer to outer wall), which may result in 12–29% oversizing [39]. The 33 and 36 mm diameters of the Colt device may increase the number of patients suitable for implantation of one endoprosthesis module, without the need for an additional wider proximal stent graft, which may reduce the risk of paraplegia.

Although the 24-F introducing system is relatively large, compared with the 22-F outer diameter of the Zenith t-Branch device which is also loaded into a 24-F sheath, it is still possible to undertake its insertion as a percutaneous procedure. The 18-F low profile t-Branch is available only as custom-made device. However, we prefer formal surgical exposure of all access sites for better control of bleeding. Some authors have used the “preclose technique” even for 24-F introducing systems [40, 41].

Having experience with the Colt and with other commercially available devices we find that major advantage of this endograft is the possibility of changing the branches in case of technical problems with cannulation and implantation of a bridging peripheral stent graft to the target vessel which can occur during the procedure. This feature of the Colt device should be taken into account in case of complex aneurysm anatomy because of its versatile shape and conformability. Even though we present only elective cases in our series, some authors suggest that this system, owing to its characteristics, may also be used as an off-the-shelf service in emergency cases [42, 43]. Angiletta *et al*. [42] presented the use of the Colt device as a rescue device after failed endovascular exclusion of ruptured aneurysm with other commercial endografts. In our center we have also used the Colt for failed EVAR and endovascular aneurysm sealing (EVAS) in several cases but have not included these patients in this series, to avoid sampling bias [44].

## 5. Evolution of the Initial Project

Since the first application of the Colt multibranched device, some modifications of the initial concept have been introduced. Different sizes of the docking station are now available. Changes include the diameters as well as the lengths of the proximal and distal part of the stent graft. The shortening of the proximal part of the graft reduces the risk of paraplegia. The Italian group pointed out that the fixed length of the Zenith t-Branch device determines a higher coverage of the nondiseased aorta compared with the custom-made devices [43]. This problem was also discussed by Ferreira *et al*. [23] who modify the Zenith t-Branch device by cutting its proximal or distal part in particular cases of supra- and juxtarenal aneurysms. Our experience indicates that once unsheathed the graft may rotate when placed in the delivery system leading to problems with proper positioning, particularly in case of complex aortic anatomy. Having various lengths of the Colt docking station at our disposal, we can use the device for these aneurysms without any modifications. The main evolutionary changes involved the branched part of the docking station. During the first three implantations some problems with the cannulation of side branches occurred. The change was introduced in the branches, which are now sewn to the main body with a separate patch each. This creates the elliptic shape of the orifice and keeps it open. This design, combined with proximal flaring of the balloon-expandable peripheral stent graft, increases the sealing force. It is worth emphasizing that we did not notice any dislocation between the branch and the balloon-expandable or self-expanding bridging stent graft.

The small number of patients, different bridging stent grafts, and modifications of the initial Colt device limit our findings. Our results encourage conducting a clinical trial potentially resulting in an off-the-shelf device.

## 6. What Makes the Colt Device Unique?

The docking station is available in two different diameters and three different lengths, which makes the endograft more universal and may reduce the risk of SCI by shortening the length of the proximal landing zone in healthy descending aorta. The nitinol struts of the device skeleton make it more conformable. All branches are directed downward, not at an angle. This may facilitate cannulation in small aortic lumens and also offers the operator the opportunity to decide which branch to choose for the target vessel even during implantation. Staged and rescue procedures are possible. The docking station matches with various devices, which allows it to be used as an off-the-shelf device also in urgent cases and for failed EVAR and EVAS.

## 7. Conclusions

This retrospective analysis shows the feasibility of a new custom-made E-xtra design stent graft device for treating TAAA. The Colt, a multibranched stent graft, may be applied in a wide variety of TAAA anatomies. Long-term follow up and a larger group of cases are needed to prove applicability of this novel device in the treatment of TAAA.
